# Ultra-Reliable Deep-Reinforcement-Learning-Based Intelligent Downlink Scheduling for 5G New Radio-Vehicle to Infrastructure Scenarios

**DOI:** 10.3390/s23208454

**Published:** 2023-10-13

**Authors:** Jizhe Wang, Yuanbing Zheng, Jian Wang, Zhenghua Shen, Lei Tong, Yahao Jing, Yu Luo, Yong Liao

**Affiliations:** 1 State Grid Chongqing Information and Telecommunication Company, Chongqing 400012, China; 2 School of Microelectronics and Communication Engineering, Chongqing University, Chongqing 400044, China

**Keywords:** 5G, NR-V2I, automatic driving, DDQL, ultra-reliable

## Abstract

Higher standards for reliability and efficiency apply to the connection between vehicle terminals and infrastructure by the fifth-generation mobile communication technology (5G). A vehicle-to-infrastructure system uses a communication system called NR-V2I (New Radio-Vehicle to Infrastructure), which uses Link Adaptation (LA) technology to communicate in constantly changing V2I to increase the efficacy and reliability of V2I information transmission. This paper proposes a Double Deep Q-learning (DDQL) LA scheduling algorithm for optimizing the modulation and coding scheme (MCS) of autonomous driving vehicles in V2I communication. The problem with the Doppler shift and complex fast time-varying channels reducing the reliability of information transmission in V2I scenarios is that they make it less likely that the information will be transmitted accurately. Schedules for autonomous vehicles using Space Division Multiplexing (SDM) and MCS are used in V2I communications. To address the issue of Deep Q-learning (DQL) overestimation in the Q-Network learning process, the approach integrates Deep Neural Network (DNN) and Double Q-Network (DDQN). The findings of this study demonstrate that the suggested algorithm can adapt to complex channel environments with varying vehicle speeds in V2I scenarios and by choosing the best scheduling scheme for V2I road information transmission using a combination of MCS. SDM not only increases the accuracy of the transmission of road safety information but also helps to foster cooperation and communication between vehicle terminals to realize cooperative driving.

## 1. Introduction

Vehicles with autonomous driving capabilities are presently advancing quite rapidly. Numerous developments and investigations have been conducted recently to enhance the capacity of connected automobiles to transmit data about their surroundings. The vehicles to everything (V2X) is a sizable interactive network made up of vehicle location information including speed and location, and it involves four different types of communication: vehicle-to-vehicle (V2V), vehicle-to-infrastructure (V2I), vehicle-to-network (V2N), and vehicle-to-pedestrian (V2P) [1]. The intelligent transportation system (ITS), which is intended to improve driving convenience and safety, includes V2I communication technology as a key component. Vehicles can receive more comprehensive road information from the infrastructure, like warnings about construction zones, traffic accidents, and traffic congestion, enabling them to make better driving judgments. In order to increase traffic efficiency and lessen congestion, the infrastructure can also alter the timing of signal lights and optimize the timing of traffic signals through communication with vehicles [2].

Users have extremely high expectations for ultra-reliable and low-latency communication (URLLC) in the V2I scenario of the Internet of vehicles, which is also essential for maintaining road safety. The Internet of vehicles and fifth-generation mobile communication technology (5G) are both developing at the same time, and NR-V2X leverages link adaptation (LA) to give URLLC more robust technical support. Through the use of the V2I channel quality adjustment modulation and coding scheme (MCS) in NR-V2X, LA may provide reliable transmission. Adaptive modulation and coding (AMC) make it possible for ITS’s intelligent vehicle communications to have better spectrum awareness ability [3].

The AMC modifies the transmission parameters in accordance with the channel’s quality at each given time. Data transmission rates can increase with faster modulation and encoding rates. If the channel conditions are bad, some transmission rates can be sacrificed to lower transmission mistakes, while the modulation methods and coding rates can be decreased to retain reliability. Fixed lookup tables, inner loop link adaptation (ILLA), outer loop link adaptation (OLLA), and no outer loop link adaptation (NoOLLA) technologies are common components of traditional AMC solutions. In the realm of AMC, OLLA technology is a higher-level adaptive technique that has the ability to dynamically modify the settings in accordance with network resources and global performance indicators. While using a predefined parameter configuration for data transmission, NoOLLA technology is a fixed method that is easier and does not require the idea of outer ring adjustment [4]. The first receiver provides feedback on the channel state information (CSI) in the conventional AMC. The transmitter then examines the channel state data to determine the correlation between the channel quality index (CQI) and the signal-to-noise ratio (SNR). The transmitter will automatically modify MCS to achieve adaptive switching based on this relationship [5]. In a V2I scenario, the vehicle’s fluctuating speed and the random scattering phenomenon in a high-speed driving environment would cause the transmission signal to travel along a number of different paths as it attempts to reach the base station (BS). Due to the separate and quick temporal phase shifts caused by the various Doppler shift on these paths, the channel rapidly fades (for instance, the amplitude and phase of the entire channel change quickly over time). In this instance, a channel quality indicator based solely on SNR has been unable to adequately depict the channel’s actual state. The effectiveness of communicating information about road safety and the throughput of data communication may suffer significantly as a result of the effects of rapid deterioration [6].

The use of machine learning (ML) technology in ITS has grown significantly in recent years [7]. The literature [8,9] discusses the use of deep learning algorithms in AMC and compares the effectiveness of algorithms, such as convolutional neural network (CNN), ResNet, DenseNet, and convolutional long and short-term deep neural network (CLDNN), in classifying signal modulation types. The ML technique of reinforcement learning (RL) has also been used for a variety of issues, such as resource optimization, coverage and capacity optimization, and backhaul optimization [10]. According to the literature [11], when using RL in AMC, the received signal to interference-plus-noise ratio (SINR) is used to determine the MCS, and because SINR is a continuous variable, the state space is similarly continuous. When dealing with such a continuous state space, this enables the learning algorithm to take a wider state space into consideration. According to the literature [12], the MCS selection rules are modified using RL algorithms in order to take into account the consequences of prior AMC judgments. According to the literature [4], based on the Q learning algorithm, BS can independently investigate and choose the best MCS schemes to maximize spectral efficiency while retaining a low bit error rate (BER). In order to help agents deal with high-dimensional state spaces, learn complex strategies, increase learning efficiency, and apply to the continuous motion space problem, deep reinforcement learning (DRL) combines the benefits of deep learning and RL [13]. Based on this, a study [14] utilizing DRL developed an intelligent MCS selection algorithm with outstanding transmission rate performance in the setting of cognitive heterogeneous networks. The Deep Q-network (DQN) algorithm is a popular one for DRL. For the joint scheduling of MCS and space division multiplexing (SDM) in the 5G massive MIMO-OFDM system, the literature [15] suggests a DQN-based approach.

Traditional DQN uses a single neural network for both action selection and Q value estimation, which leads to an excessive Q value estimate [15]. Two neural networks are introduced by the double deep Q-network (DDQN), one for action selection and the other for Q value estimation [16]. By choosing an action and assessing its Q value at each update, this dual-network structure can decrease the overestimation of Q value and improve the stability and performance of DDQN [17]. Therefore, in order to improve the performance of the DQN-based scheduling algorithm in the literature [14] and make it more adapted to ultra-reliable intelligent downlink scheduling, this paper suggests a massive MIMO intelligent scheduling technique based on DDQN for the 5G NR-V2I scenario. This approach is employed for intelligent joint scheduling of MCS, precoding matrix indicator (PMI), and SDM. This paper suggests a highly trustworthy intelligent downlink scheduling technique based on DDQN for the 5G NR-V2I scenario. The following are its specific contributions:

(1) Eliminate the overvaluation issue with Q value—when learning the Q value function for the DQN algorithm, the Q value is prone to being overstated, which means that for some state–action combinations, its Q value might overestimate. Due to this, the DQN algorithm may occasionally choose ineffective actions, which will have an impact on the scheduling efficiency. The overestimation problem of Q values can be reduced by DDQN by using two Q networks, one for choosing actions and the other for assessing the value of those activities, therefore enhancing the precision and stability of downlink scheduling algorithm learning.
(2) More precise action choice—dual Q networks are utilized by the DDQN algorithm to pick activities, which allows for a more precise assessment of the relative worth of various actions. Due to this, DDQN may be able to choose actions with greater precision, improving the downlink scheduling approach. The DDQN algorithm can more precisely choose the actions that can optimize throughput or lower the BER, thereby enhancing link performance, when compared to DQN, OLLA, and NoOLLA.
(3) Overcoming the issue of the local optimal solution—the OLLA algorithm may enter the local optimal solution and fail to attain the global optimal by optimizing the local action selection. The DDQN algorithm, in contrast, employs dual Q networks throughout the learning phase, which can better avoid the local optimal solution problem and more effectively explore the larger action space.
(4) Adapt to surroundings that are more complicated—by using two Q networks and reinforcement learning, the DDQN algorithm can adapt more flexibly to various channel environments and network requirements under a dynamic, changing environment, so as to improve the efficiency and reliability of communication links. This makes DDQN have strong adaptability and superior performance in a complex environment.

This paper is organized as follows. The downlink adaptive scheduling model based on the channel-state information reference signal (CSI-RS) is primarily established in Section 2. The adaptive technique of V2I downlink scheduling based on DDQN is introduced in Section 3, along with the measurement of the downlink channel, data processing, network architecture, and training parameter setup. In Section 4, the simulation results are verified. The conclusion is provided in Section 5.

## 2. Problem Formulation

**Figure 1:**
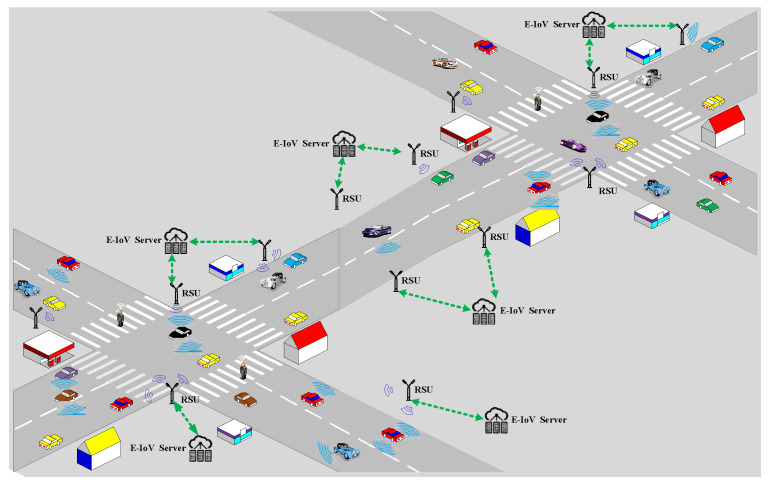
Communication scenarios for NR-V2I.

Through the policy modification of the downlink communication, NR-V2I improves the communication reliability and spectrum efficiency of the vehicle terminal. The application scenario of NR-V2I [18] is given in Figure 1. A lower modulation scheme and coding rate can be utilized when the edge-Internet of vehicles (E-IoV) server delivers signals to the vehicle terminal through the road side unit (RSU), which will boost the robustness for weak connections. In addition, the E-IoV Server increases spectral efficiency (SE) by using a higher modulation scheme and coding rate.

**Figure 2:**
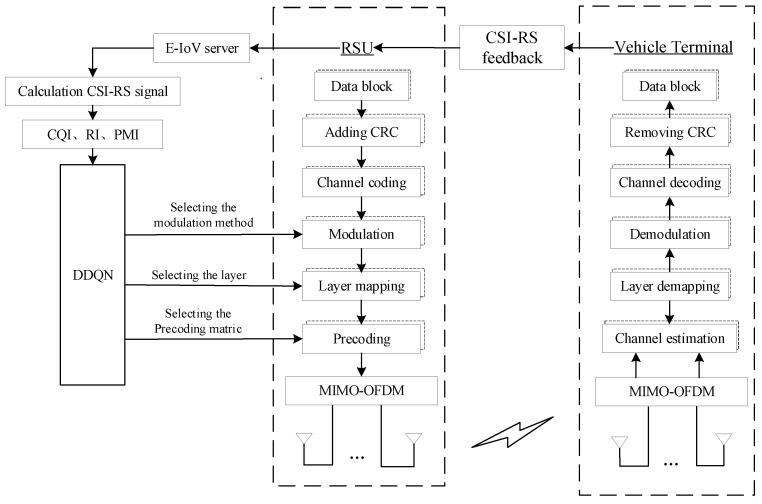
Scheduling for NR-V2I communication systems with reliable links.

The MIMO-OFDM communication system of NR-V2I [19] (Individual User 1) is used as the research subject in this work. The intelligent link scheduling approach based on DRL is used in the downlink adaptive scheduling of CSI-RS. In Figure 2, the scheduling is displayed. The fundamental principles of NR-V2I communication are as follows.

The vehicle terminal measures the CSI-RS sent from the RSU side and then feeds the signal back to the RSU through the physical uplink data channel. The E-IoV server chooses the downlink scheduling scheme based on the feedback value of the CSI-RS transmitted by the RSU, which provides an ultra-reliable and low-latency communication scheme for the current data transmission of in-vehicle terminals through the DDQN method.

Consider how [20] may be employed to describe the channel capacity in a MIMO context.

(1) $$V(H)=\log_{2}\left\{\det\left[E_{N_{r}}+\eta \left(WH\right){\left(WH\right)}^{H}\right]\right\}$$

where V is the channel capacity; $H\in {\mathbb{C}}^{N_{r}\times N_{t}}$ is the channel matrix; $N_{t}$ and $N_{r}$ are, respectively, the number of transmitting and receiving antennas; the letter $E_{N_{r}}$ stands for the unit matrix in $N_{r}$ dimensions; η denotes the signal transmitting power to noise power ratio; W is the beam fugitive matrix; ${\left(\cdot \right)}^{H}$ indicates the conjugate transpose matrix of the solver matrix; and $\det\left(\cdot \right)$ is the solver matrix’s determinant. The RSU’s downlink adaptive scheduling, which is closely connected to the RSU’s downlink adaptive scheduling, has a significant impact on the BER of the real downlink of the NR-V2I communication system.

The code elements in the NR-V2I communication system are encoded in an OFDM resource block (RB) for cyclic redundancy check (CRC), and if the check is unsuccessful, all of the RB’s code elements are retransmitted. You may obtain the downlink BER $B_{\mathrm{slot}}$ for a single time slot by:

(2) $$B_{\mathrm{slot}}=B_{e}/(l\cdot c\cdot m\cdot N_{\mathrm{RB}}\cdot N_{\mathrm{RE}})$$

where Be refers to the number of downlink transmission error bits; l is the number of downlink-scheduled layers for air-division multiplexing; c is the number of downlink-scheduled bits for data transmission code; m indicates the number of modulated downlink-scheduled data symbols; $N_{\mathrm{RB}}$ denotes the number of downlink-scheduled resource blocks (RBs); and $N_{\mathrm{RE}}$ is in the name of the number of resource blocks (Res) that make up each RB. When the subcarrier spacing is 15 kHz, there are 14 OFDM symbols and 12 subcarriers in one RB in OFDM. $N_{RB}$ and $N_{\mathrm{RE}}$ are treated as fixed values in this paper. They primarily depend on the resource allocation and are independent of the link-adaptive downlink scheduling policy.

A mathematical description of the downlink adaptive scheduling method based on the CSI-RS may be obtained from (3):

(3) $$\underset{B_{e},\;P_{\mathrm{SE}}}{\arg\min}B_{\mathrm{slot}}=Be/(P_{SE}N_{RB}N_{RE})$$

(3a) $$s.t.\;P_{SE}=l\cdot P_{U\text{-}\mathrm{SE}}=f\left(D_{\mathrm{CQI}},D_{\mathrm{RI}},D_{\mathrm{PMI}},B_{P\text{-}\mathrm{slot}}\right)$$

(3b) 1≤l≤4

(3c) $$P_{U\text{-}SE}=c\cdot m=M\left(D_{\mathrm{MCS}}\right)$$

(3d) m∈{1,4,6,8}

The intention of the downlink adaptation based on the CSI-RS is to reduce the BER. Bslot represents the number of incorrect bits following the current time slot scheduling. The state variables are the CQI,RI and PMI determined by the E-IoV Server based on the CSI-RS fed back from the vehicle terminals delivered by the RSU and the BER $B_{P\text{-}\mathrm{slot}}$ obtained through statistics after the prior time slot has been scheduled. The decision variables l are and $D_{\mathrm{MCS}}$.

(4) $$P_{\mathrm{SE}}=r\cdot P_{U\text{-}\mathrm{SE}}=l\cdot M\left(D_{\mathrm{MCS}}\right)=f\left(D_{\mathrm{CQI}},D_{\mathrm{RI}},D_{\mathrm{PMI}},B_{P\text{-}\mathrm{slot}}\right)$$

where, as indicated in Equation (4), the spectral efficiency is $P_{\mathrm{SE}}$. Furthermore, the $D_{\mathrm{CQI}}$, $D_{\mathrm{RI}}$ and $D_{\mathrm{PMI}}$ stand for, respectively, the CQI, RI and PMI calculated by the E-IoV server. $f\left(\cdot \right)$ stands for the downlink adaptive scheduling algorithm based on the CSI-RS, with the SEs discounted by the l and $D_{\mathrm{MCS}}$ as their outputs. The algorithm’s inputs are the CQI,RI,PMI and Be supplied by the E-IoV server.

In Equation (5), $P_{U\text{-}\mathrm{SE}}$ stands for Unit-Spectral Efficiency, or U-SE.

(5) $$P_{U\text{-}\mathrm{SE}}=l\cdot m=M\left(D_{\mathrm{MCS}}\right)$$

where the $M\left(D_{\mathrm{MCS}}\right)$ function represents the U-SE acquired at a certain order $D_{\mathrm{MCS}}$ that corresponds to the current order. The primary scheduling parameters produced by the downlink adaptive method are the number of downlink air-division multiplexing layers l, the downlink data coding rate c, and the downlink symbol modulation order m. $\left(c\cdot m\right)$ symbolizes the number of bits that are acceptable on a single RE.

When the scheduling of l and $D_{\mathrm{MCS}}$ grows more than the current channel conditions of the vehicle terminal support demodulation capacity, Be and $B_{\mathrm{slot}}$ shall grow. The downlink space division multiplexing layer number l and MCS order $D_{\mathrm{MCS}}$ two parameters primarily reflect the transmission data density. In addition, even when Be is reduced, the system’s $B_{\mathrm{slot}}$ will not reach the minimum value of $B_{\mathrm{slot}}$ due to the excessively conservative amount of scheduling data when l and $D_{\mathrm{MCS}}$ scheduling tend to be significantly less than the demodulation capability supported by the vehicle terminal under the current channel conditions. With the goal of bringing the system into balance with the Be while minimizing the system’s $B_{\mathrm{slot}}$, the number of layers l of downlink space division multiplexing and the order of MCS $D_{\mathrm{MCS}}$ scheduling must be closely matched to the current channel state and the demodulation capability of the vehicle terminals.

## 3. DDQN-Based V2I Downlink Scheduling Adaptation

### 3.1. Downlink Channel Measurement

For the purpose of downlink channel measurement in the NR-V2I communication system depicted in Figure 2, the RSU periodically inserts the CSI-RS into the downlink data frame and then transmits it to the onboard terminal. The scheduling strategy for the downlink will ultimately be influenced by the measuring results of the feedback from the onboard terminal to the RSU. If the onboard terminal has $N_{r}$ receiving antennas and $N_{t}$ transmitting antennas at the RSU, and the signal flow during transmission is described as

(6) $$y_{\mathrm{CSI}\text{-}\mathrm{RS}}=S_{\mathrm{CSI}\text{-}\mathrm{RS}}h_{\mathrm{DL}}+n_{\mathrm{DL}}$$

the remaining ports transmit zero pilot because the CSI-RS is mapped to various time-frequency domain positions on various transmitting antennas. We can therefore infer that CSI-RS per transmitting antenna is:

(7) $$S_{\mathrm{CSI}\text{-}\mathrm{RS}}=\mathrm{diag}\left(s_{\mathrm{CSI}\text{-}\mathrm{RS}}\right)$$

The emitted CSI-RS vector can be expressed as $s_{\mathrm{CSI}\text{-}\mathrm{RS}}={\left[\begin{array}{cccccc} s_{1} & s_{2} & \ldots & s_{q} & \ldots & s_{N_{r}} \end{array}\right]}^{T}$ because $\mathrm{diag}\left(\cdot \right)$ indicates building $s_{\mathrm{CSI}\text{-}\mathrm{RS}}$ as a diagonal matrix. The CSI-RS vector of each transmitting antenna to the receiving antenna q may be expressed as $s_{q}=\left[\begin{array}{cccccc} s_{q,1} & s_{q,2} & \ldots & s_{q,p} & \ldots & s_{q{,N}_{t}} \end{array}\right]$.

The received CSI-RS vector is expressed as $y_{\mathrm{CSI}\text{-}\mathrm{RS}}={\left[\begin{array}{cccccc} y_{{}_{1}}^{T} & y_{{}_{2}}^{T} & \ldots & y_{q}^{T} & \ldots & y_{{}_{N_{r}}}^{T} \end{array}\right]}^{T}$; however, the CSI-RS vector received by the receiving antenna may be expressed as $y_{q}=\left[\begin{array}{cccccc} y_{q,1} & y_{q,2} & \ldots & y_{q,p} & \ldots & y_{q,N_{t}} \end{array}\right]$. Additionally, the channel response on the receiving antenna q is represented as $h_{q}=\left[\begin{array}{cccccc} h_{q,1} & h_{q,2} & \ldots & h_{q,p} & \ldots & h_{q,N_{t}} \end{array}\right]$; hence, the downstream channel’s channel response is $h_{\mathrm{DL}}={\left[\begin{array}{cccccc} h_{{}_{1}}^{T} & h_{{}_{2}}^{T} & \ldots & h_{q}^{T} & \ldots & h_{{}_{N_{r}}}^{T} \end{array}\right]}^{T}$. A noise vector $\delta_{n}^{2}$ with a mean of 0 and a variance of $n_{\mathrm{DL}}\in {\mathbb{C}}^{N_{t}N_{r}\times 1}$ is then used to represent the noise on the channel.

Formula (6) and the CSI-RS of each transmitting antenna allow for the least square (LS) estimation of the downstream channel response vector ${\hat{h}}_{\mathrm{DL}}$:

(8) $${\hat{h}}_{\mathrm{DL}}={\left(S_{\mathrm{CSI}\text{-}\mathrm{RS}}\right)}^{-1}y_{\mathrm{CSI}\text{-}\mathrm{RS}}$$

Additionally, obtain the downlink channel response matrix ${\hat{H}}_{\mathrm{DL}}$:

(9) $${\hat{H}}_{\mathrm{DL}}=\left[\begin{array}{cccc} {\hat{h}}_{1}^{T} & {\hat{h}}_{2}^{T} & \ldots & {\hat{h}}_{N_{r}}^{T} \end{array}\right]=\left[\begin{array}{cccc} {\hat{h}}_{1,1} & {\hat{h}}_{2,1} & \cdots & {\hat{h}}_{N_{r},1}\\ {\hat{h}}_{1,2} & \ddots & & \vdots \\ \vdots & & \ddots & \vdots \\ {\hat{h}}_{{1,N}_{t}} & \cdots & \cdots & {\hat{h}}_{N_{r}{,N}_{t}} \end{array}\right]$$

Then, the onboard terminal will obtain the RI, PMI, and CQI based on the estimated ${\hat{H}}_{\mathrm{DL}}$ measurement and feed the above measurements back to the RSU. The vehicle terminal will be based on the estimated ${\hat{H}}_{\mathrm{DL}}$. The value of RI is usually related to the number of antennas and the channel environment, and higher RI values indicate better space fraction multiplexing capability. The eigenvalues of the channel matrix are obtained by performing an eigenvalue decomposition of the channel matrix ${\hat{H}}_{\mathrm{DL}}$.

(10) $${\hat{H}}_{\mathrm{DL}}^{}=U_{\mathrm{DL}}\Sigma_{\mathrm{DL}}V_{\mathrm{DL}}^{H}$$

In particular, the eigenvalues $\Sigma_{\mathrm{DL}}$ reflect the singular values of the channel, which reflect the capacity of the channel to transmit signals across its many layers. $U_{\mathrm{DL}}$ and $V_{\mathrm{DL}}$ are unitary matrices. Consequently, the RI can be determined using the following equation:

(11) $$D_{\mathrm{RI}}=\left\{\begin{array}{cc} \;0\;\; & if\;\;Z\left(\Sigma_{\mathrm{DL}}-N_{\mathrm{DL}}\right)=0,1\\ Z\left(\Sigma_{\mathrm{DL}}-N_{\mathrm{DL}}\right) & else \end{array}\right.$$

The $Z\left(\cdot \right)$ function determines the number of diagonal elements in the matrix that are greater than zero, where $N_{\mathrm{DL}}=\delta_{n}^{2}I_{N_{r}}$ is the noise matrix of each layer.

When RI values are known, they can be mapped to the corresponding precoded matrix index using predefined PMI tables [21]. The collection of possible PMI matrices is $S_{\mathrm{PMI}}$, and the values of $N_{t}$, $N_{r}$ and $D_{\mathrm{RI}}$ are known. If the PMI matrix corresponding to the PMI matrix index $D_{\mathrm{PMI}}$ is $W_{D}{}_{{}_{\mathrm{PMI}}}$, $W_{D}{}_{{}_{\mathrm{PMI}}}\in S_{\mathrm{PMI}}$, it will assume that element $S_{\mathrm{PMI}}$ has $N_{\mathrm{PMI}}$ elements. The estimated SNR matrix $\Gamma_{D_{\mathrm{PMI}}}$ can be computed using the downlink precoding matrix $W_{D}{}_{{}_{\mathrm{PMI}}}$, as follows:

(12) $$\Gamma_{i_{\mathrm{PMI}}}={\left[N_{DL}{\left(W_{D_{\mathrm{PMI}}}^{}{\hat{H}}_{\mathrm{DL}}^{H}{\hat{H}}_{\mathrm{DL}}^{}W_{D_{\mathrm{PMI}}}^{H}+N_{DL}\right)}^{-1}\right]}^{-1}$$

To fully account for the influences between multiple levels, the SNRs of each layer were merged to obtain an integrated SNR value. A second norm of $\Gamma_{D_{\mathrm{PMI}}}$ can be used to produce the combined SNR $\rho_{\mathrm{PMI}}$. There are various PMI matrices available in the collection of PMIs, each of which corresponds to a distinct precoding technique. Because there are fewer aggregate elements, the onboard terminal can poll (or traverse) each PMI index in turn and determine the appropriate combined SNR value. The merged SNR value for each candidate PMI index was calculated, and the PMI index that maximizes the SNR value was then identified. The onboard terminal returns the index to the RSU in the following manner after locating the ideal PMI index:

(13) $$\underset{D_{\mathrm{PMI}}}{\mathrm{argmax}}\begin{array}{c} \rho_{\mathrm{PMI}} \end{array}={\left\|\Gamma_{D_{\mathrm{PMI}}}\right\|}_{F}^{2}$$

The CQI is a channel quality indicator that is frequently used in communication systems for adaptive modulation and encoding [22]. The mapping function $D_{\mathrm{CQI}}$ can be used to determine the appropriate CQI index $M_{\mathrm{CQI}}\left(\cdot \right)$ for the decibel representation of $\rho_{\mathrm{PMI}}$:

(14) $$D_{\mathrm{CQI}}=M_{\mathrm{CQI}}\left(\log_{2}\left(\rho_{\mathrm{PMI}}-1\right)\right)$$

The onboard terminal will now encode the RI, PMI, and CQI measured data into a feedback signal and transmit them back to the RSU. The RSU will decide the downlink scheduling choice method based on the aforementioned facts after receiving this report.

### 3.2. Data Processing and Network Architecture

**Figure 3:**
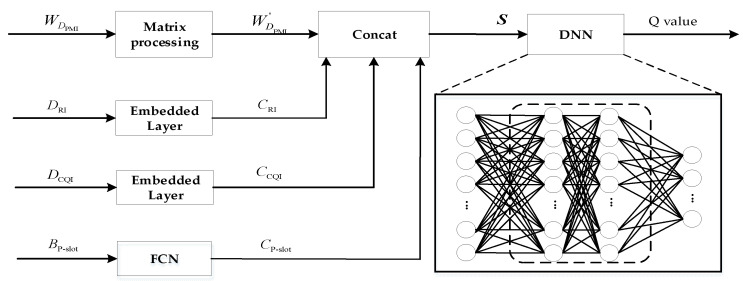
Schematic diagram of DDQN network structure.

The direct application of the DQN algorithm will end up resulting in an overestimation of the decision value [23] due to the complexity of the NR-V2I communication system, the analog nature of the states and actions, and the volume of data. As a result, in this paper, we use the DDQN for downlink scheduling and the DNN network for calculating the Q value rather than the Q-Table. Figure 3 depicts the DDQN’s structural layout.

Figure 3 depicts the network structure used in DDQN. It primarily consists of the data preprocessing section, the Concat layer, and the DNN layer. The DNN layer is a Full Convolutional Neural Network (FCN), where the input is the current state S and the output is the Q value of the reward value corresponding to all of the actions in the current state.

The $D_{\mathrm{CQI}}$, $D_{\mathrm{RI}}$ and $D_{\mathrm{PMI}}$ from the measurement feedback of the vehicle terminal, as well as the statistically obtained $B_{P\text{-}\mathrm{slot}}$, are the primary sources of information for the DDQN used in this paper to output downlink adaptive scheduling. Because the dimensionality of each variable varies, it is necessary to preprocess the data before inputting them into the DNN network. The preprocessing of input data to the DNN network consists of the following parts:

1. Matrix processing: Equation (15) illustrates how one may acquire the precoding matrix $W_{D_{\mathrm{PMI}}}\in {\mathbb{C}}^{N_{t}\times N_{r}}$ for the precoding matrix and obtain $W_{D_{{}_{\mathrm{PMI}}}}^{\prime }\in {\mathbb{R}}^{2N_{t}\times N_{r}}$ following the same matrix processing:

(15) $$W_{D_{{}_{\mathrm{PMI}}}}^{\prime }=\left[\begin{array}{l} Re(W_{D_{\mathrm{PMI}}})\\ Im(W_{D_{\mathrm{PMI}}}) \end{array}\right]$$

where Re(⋅) and Im(⋅) are shown as taking, respectively, the real part function and the imagistic part function.

2. Embedding layer: As a result of $D_{\mathrm{CQI}}=(0,15)\in Z,D_{\mathrm{RI}}=(0,3)\in Z$, the CQI encoding vector and RI encoding vector must be obtained to satisfy the network input conditions. These vectors can be obtained by the embedding layer network in deep learning, and the embedding layer can transform the input’s index value into a vector of a specific dimension size. The embedding layer, in particular, is essentially made up of several fully connected networks, but it has a different focus. The output of the embedding layer is equivalent to the weights in the fully connected network, which acquires the network weights.

Given that there are 16 and 4 CQI and RI values in this research, respectively, and that each coding vector possesses a dimension of $N_{r}$, the embedding matrix may be represented as follows:

(16) $$E_{CQI}={\left[e_{1}^{CQI},e_{2}^{\mathrm{CQI}},\cdots ,e_{16}^{\mathrm{CQI}}\right]}^{T}$$

(17) $$E_{RI}={\left[e_{1}^{RI},e_{2}^{RI},\cdots ,e_{4}^{RI}\right]}^{T}$$

where $E_{CQI}\in {\mathbb{R}}^{16\times N_{r}}$ and $E_{RI}\in {\mathbb{R}}^{4\times N_{r}}$. Before training, the data in the embedding matrix are set up at random. During training, the embedding layer can obtain the specified row vector in the embedding matrix as the coding vector according to the given input index value by simply applying the values of $D_{\mathrm{CQI}}$ and $D_{\mathrm{RI}}$, whose expressions are, respectively:

(18) $$C_{\mathrm{CQI}}=S(E_{CQI},D_{\mathrm{CQI}})$$

(19) $$C_{\mathrm{RI}}=S(E_{\mathrm{RI}},D_{\mathrm{RI}})$$

where $D_{\mathrm{CQI}}\in {\mathbb{R}}^{1\times N_{r}}$ and $C_{\mathrm{RI}}\in {\mathbb{R}}^{1\times N_{r}}$ are the CQI encoding vector and the RI encoding vector under the input $D_{CQI}$ and $D_{RI}$ values, respectively; $S\left(\cdot \right)$ indicates that the specified row vector in the matrix is picked as the encoding vector based on the index value.

3. Fully Connected Layer: To be able to obtain the mapping vector $C_{P\text{-}\mathrm{slot}}\in {\mathbb{R}}^{1\times N_{r}}$ of BER, high-dimensional mapping will be executed by applying the FCN network’s $B_{P\text{-}\mathrm{slot}}$ because $B_{P\text{-}\mathrm{slot}}=(0,1)\in Q$.
4. Concat operation: Following the previously mentioned process, the processed data must be concatenated into a single dimension to receive the DNN layer’s input.

(20) $$S=Concat\left(\begin{array}{l} W_{D_{{}_{\mathrm{PMI}}}}^{\prime }\\ D_{\mathrm{CQI}}\\ D_{\mathrm{RI}}\\ D_{P\text{-}\mathrm{slot}} \end{array}\right)\in {\mathbb{R}}^{{(3+2N}_{t})\times N_{r}}$$

where $Concat\left(\cdot \right)$ denotes the splicing function and S is the input to the DNN layer.

In this paper, the basic elements of the Q-learning algorithm in a DDQN system are represented as:

(1) Environment (environment): communication system with adaptive scheduling for NR-V2I downlink;
(2) Intelligent body (agent): vehicle-mounted terminal;
(3) Action: the quantity of space division multiplexing layers RI and MCS used by downlink scheduling by RSU, which is referred to as action $a=(r,D_{MCS})$ in DDQN;
(4) State: states are defined as those that are explicitly specified, as indicated in Equation (20), such as the $D_{\mathrm{CQI}}$ acquired from downlink measurement, the precoding matrix $W_{D_{\mathrm{PMI}}}$ corresponding to $D_{\mathrm{PMI}}$ and $D_{RI}$, and the state matrix S produced from $B_{P\text{-}\mathrm{slot}}$ after data preprocessing;
(5) Reward: B, which is specified as indicated in Equation (5), is defined as the BER following downlink adaptive scheduling.

**Figure 4:**
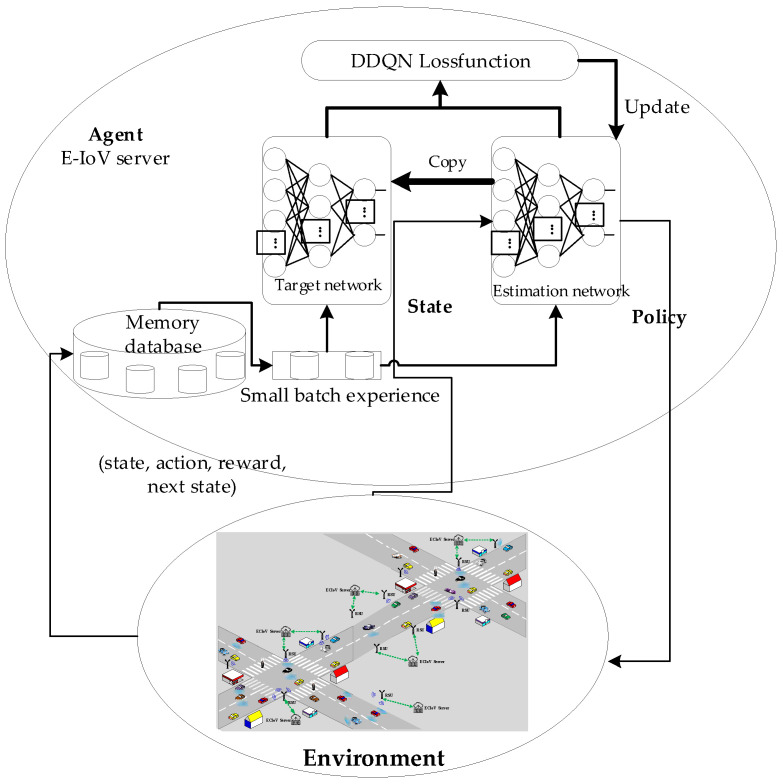
Reliable link scheduling structure based on DDQN.

A neural network is utilized to estimate the Q value rather than a Q-Table in the downlink scheduling technique based on DDQN, which was created by fusing the DNN network illustrated in Figure 4 with the Q-learning algorithm. The problem of overestimation in DQN is resolved by the reinforcement learning technique known as DDQN by splitting the computation of the desired Q value into two steps: action selection and value evaluation. The overestimation issue in DQN is resolved by DDQN, a reinforcement learning technique, by splitting the computation of target Q values into two steps: action selection and value evaluation. A memory database is inherited by the DQN to solve the relevance problem of consecutive samples. The memory database stores past experiences, such as a specific number of (state, action, reward, and next state) sample data acquired in the setting of the NR-V2I communication system, and it randomly selects a small batch of sample data to train the network in the training phase. This enables a more effective training of the DNN by using both the old and new data.

A nonlinear approach is used in DDQN to represent the Q estimator function Q(S,a;θ), where ***θ*** is a parameter of the neural network, and then the loss function in the DNN network is defined as:

(21) $$L(\theta )=E{[Q^{+}(S,a)-Q(S,a;\theta )]}^{2}$$

The parameter update of the neural network can be expressed as:

(22) $$\theta^{\left(i+1\right)}\leftarrow \theta^{\left(i\right)}-\delta \nabla L(\theta^{\left(i\right)})$$

Both the computational network and the target network are neural networks. However, they have distinct parameters while sharing the same topology. The Q-estimated value of Q(S,a) for the current state–action pair is generated by the computational network, which uses the most recent parameters. The Q-estimated value of $Q^{+}(S,a)$ is used to assess the DDQN loss function under the current channel condition–downlink scheduling mode. The target network does not update the parameters in real time, instead copying them from the computational network to the target network every specific iteration step c during the training time. Backpropagation and stochastic gradient descent (SGD) methods can be used to change the network parameters. DDQN loss function occurs under the current channel condition–downlink scheduling strategy. When the system is in the current channel uplink and downlink measurement state matrix S, the optimal state–action reward function Q(S,a) in the downlink scheduling model, indicates the largest cumulative discount gain of completing scheduling action $a^{\prime }$ to enter the next state, $S^{\prime }$. The revised phrase is written as follows:

(23) $$Q(S,a)\leftarrow Q(S,a)+\delta [r(S,a)+\gamma Q(S^{\prime },\max_{a^{\prime }}Q(S^{\prime },a^{\prime }))-Q(S,a)]$$

where γ=(0,1)∈Q stands for the pace at which future incentives will diminish and δ=(0,1)∈Q represents the learning rate. A computational network is utilized to implement the downlink adaptive scheduling procedure for NR-V2I communication once the network has been trained.

The DDQN-based downlink scheduling algorithm in this paper is shown in Algorithm 1:
**Algorithm 1:** Intelligent DDQN-based link scheduling algorithm for NR-V2I**Input:** Calculate network weights θ; target network weights $\hat{\theta }=\theta$. **Initialization:** Memory database size N; Step 1: Repeat the number of iterations episode=1 toM do;  Step 2: Initialize the state $S_{1}$;  Step 3: for the number of subframes t=1 to F do;  Step 4: The action $a_{t}$ that fulfills $a_{t}=\arg\max_{a}Q(S_{t},a;\theta )$ with probability ε, or the number of air division multiplexing layers r and the order $D_{\mathrm{MCS}}$ of MCS, is chosen by the E-IoV server;  Step 5: E-IoV server schedules the corresponding number of layers r and the order of the MCS $D_{\mathrm{MCS}}$ for the downlink, and then calculates the reward value BER $B(S_{t},a_{t})$, and the system enters a new state $S_{t+1}=S_{t}^{\prime }$;  Step 6: The memory database stores the previous iteration experience $\left(S_{t},a_{t},B(S_{t},a_{t}),S_{t}^{\prime }\right)$;  Step 7: Randomly select a small batch of sample data $\left(S_{t},a_{t},B_{t},S_{t}^{\prime }\right)$ from the memory database and train the network; the target network obtains Q target value $Q^{+}(S,a)$, and the computational network obtains Q estimated value Q(S,a);  Step 8: If the final state is reached;  Step 9: Then $Q^{+}(S,a)=r(S_{t},a_{t})$;  Step 10: Otherwise, $Q^{+}(S,a)=r(S,a)+\gamma Q(S^{\prime },\max_{a^{\prime }}Q(S^{\prime },a^{\prime }))$, γ is the decay rate of future rewards.  Step 11: Calculate the loss function according to Equation (21) and update the weights of the computational network according to Equation (22);  Step 12: Every certain number of iterations, update the parameters of the target network with the parameters of the computational network, setting $\hat{\theta }$ to $\hat{\theta }=\theta$;  Step 13: end;  Step 14: until the iteration termination condition is reached. **Output:** DDQN downlink adaptive scheduling model.

### 3.3. Training Parameter Settings

**Figure 5:**
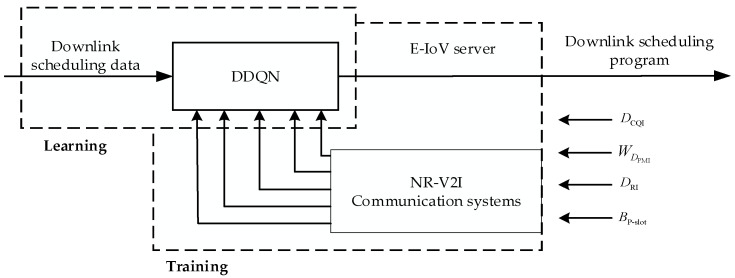
DQN-based reliable link scheduling framework.

The structure of the online learning and offline deployment phases of the DRL-based intelligent link scheduling method for NR-V2I cooperation is depicted in Figure 5.

Offline learning phase: The core of DDQN is training the neural network. To make the DDQN model applicable to various scenarios, sample downlink adaptive data from the NR-V2I communication system under various scenarios and parameters must be obtained. The DDQN model is then trained using these sample data.

This work considers two prominent cases—NR-V2I high-speed movement scenarios and scenarios with significant noise interference—where the performance of standard methods is more constrained for training and learning. Two different vehicle terminal moving speeds are taken into consideration during the training process, and the data sets of these speeds are (60 km/h, 120 km/h), which used to train the DDQN downlink adaptive network for high-speed mobile scenarios. Different delay value data sets are also given consideration, with configured delays ranging from 0 to 15 with a step size of 1. The NR-V2I communication environment must be represented in an appearance that is consistent with the reinforcement learning environment in order to apply reinforcement learning techniques to the downlink adaptation challenge.

In this paper, the NR-V2I communication environment is constructed by using the matlab platform, and pytorch, an open-source deep learning framework, is employed to build and deploy the reinforcement learning component. The interaction between the data and the environment may be realized by using the python and matlab platforms. The training process can be described as a continuous interaction between the intelligent body and the environment for the intelligent body to choose the best course of action. An Intel(R) Xeon^®^E5-2678V3 CPU with 64 GB of RAM, an NVIDIA GeForce RTX2080Ti graphics card, and Python 3.9 and Pytorch 1.13 deep learning framework serve as the hardware and software platforms for the training. The training settings for the DQN system and the simulation parameters for the NR-V2I communication system are specified as indicated in Table 1 and Table 2, respectively.

**Figure 6:**
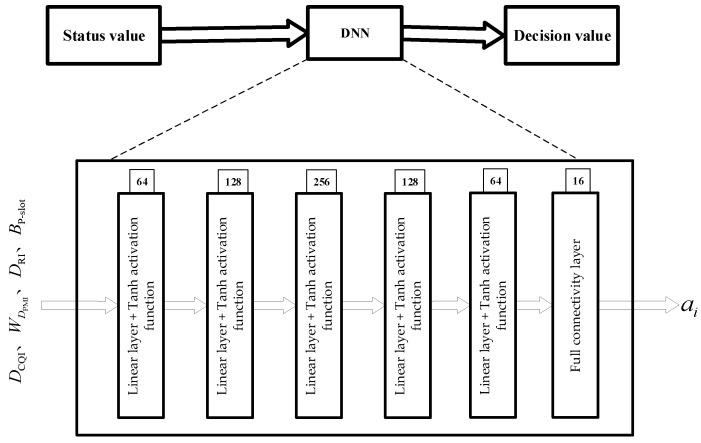
DNN layer network structure.

The DNN is an input layer with σ nodes that are connected to the components of S; there are five hidden layers with 64, 128, 256, 128, and 64 nodes, respectively; each hidden layer has a Tanh activation function; and there is an output layer with τ nodes. The structure is shown in Figure 6, where $a_{i}$ denotes the value of the optimal downstream scheduling plan that the DNN has obtained. The last layer of the output adopts a fully connected layer, and the number of output nodes corresponds to the quantity of communication decisions given by the E-IoV server to the vehicle terminal.

In this paper, the learning rate is specified in this study to be 0.01, and the future reward decay γ is specified to be 0.9. The modulation methods employed in the present investigation are QPSK, 16 QAM, 64 QAM, and 256 QAM. The channel model is the tapped delay line (TDL). The Adaptive Moment estimation (ADAM) technique, which can adaptively update the learning rate and SGD, can be employed to update the network parameters of the DQN network. The training of the network occurs when the sample data in the memory database reach 300 and continues until the network converges. A batch size of 16 indicates that 16 sample data are randomly selected from the memory database for training each time. The DQN network outputs the BER magnitude for all downlink transmission modes after network training is complete. The RSU then chooses the MCS and the number of air-division-multiplexing layers that, through the Q-learning principle, will yield the BER that is most suitable for downlink communication.

## 4. Simulation Results and Analysis

**Figure 7:**
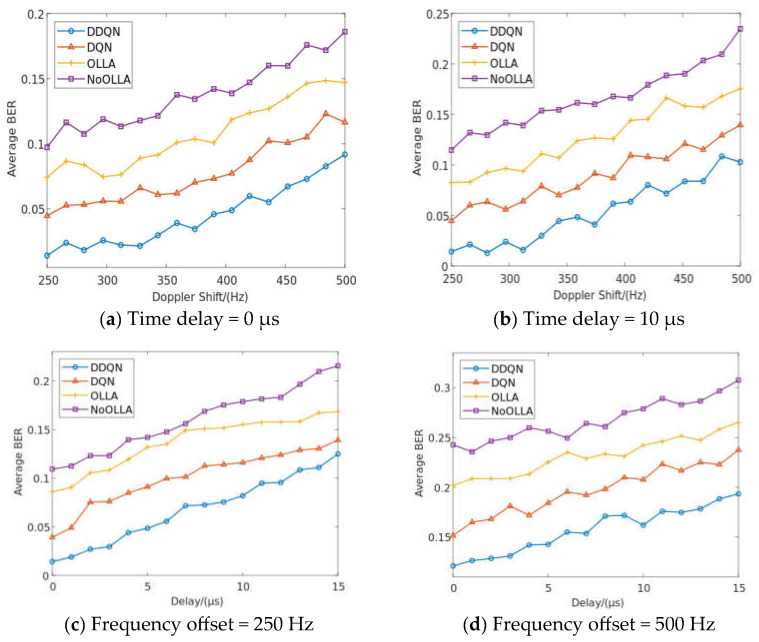
Average BER performance of different algorithms in high-speed moving scenarios.

**Figure 8:**
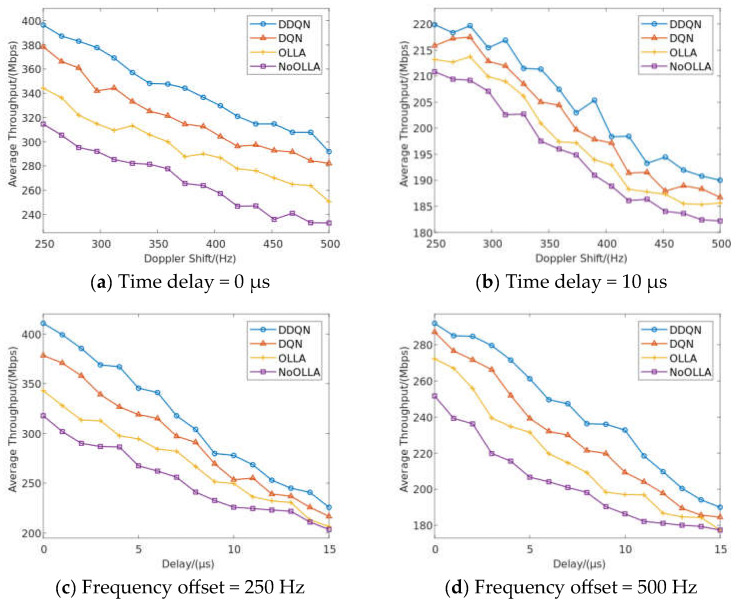
Throughput performance of different algorithms in high-speed mobility scenarios.

In this section, we compare the proposed algorithm to the OLLA, DQN, and NoOLLA algorithms in a typical high-speed moving scenario in order to assess how well the proposed algorithm performs in terms of average BER and throughput when used to schedule highly reliable intelligent downlinks in a 5G NR-V2I scenario. After simulating the algorithm using the primary communication system and DDQN network characteristics as described in Table 1 and Table 2, Figure 7 and Figure 8 display the simulation results for the average BER and throughput. Last, we compare the average number of iterations between DQN and DDQN.

In a 5G NR-V2I scenario, the vehicle often needs high data transmission reliability, particularly for security-related data transmissions, like traffic information and vehicle state updates. Because of the algorithm’s low average BER performance, it may effectively lower the error rate of data transmission even when there is a high signal-to-noise ratio and a complex channel, increasing the dependability of data transmission. Signals may experience multiple path propagation in high-speed movement circumstances, leading to multipath effects. Signals can interpolate due to multipath effects, increasing the likelihood of intersymbol interference (ISI) and raising the BER. Different frequency components can result from high-speed movement due to selective fading of the signal at the frequency. This increases the BER of signal transmission and results in frequency-selective distortion. The BER performance of the methods at the same delay when the delay is in 0 or 10 μs is shown in Figure 7a,b, respectively. The suggested method is 0.05, 0.07, and 0.1 lower than the average BER using DQN, OLLA, and NoOLLA, respectively, when the delay and frequency bias are 0 us and 436 Hz. The average BER performance of several algorithms under doppler shifts of 250 Hz and 500 Hz, respectively, is shown in Figure 7c,d. In particular, the suggested DDQN method greatly improves the average BER performance at the same multispectral frequency shift. The suggested algorithm is 0.04, 0.08, and 0.1 lower than the average BER using the DQN algorithm, OLLA algorithm, and NoOLLA algorithm, respectively, when the frequency bias and time delay are 250 Hz and 9 μs, respectively. Continuous action space issues can be handled with the OLLA algorithm. In order to avoid the complexity of directly searching for globally optimal actions, it separates the continuous action space into discrete local action spaces and employs local action selectors to choose actions. In contrast, using the continuous action space directly instead of the OLLA method typically entails spending more time and processing resources looking for global optimal actions. In order to develop better scheduling strategies in the high-dimensional state space and complex continuous action space of high-speed moving scenes, the OLLA algorithm can converge more quickly when compared to the NoOLLA algorithm.

The type of action space may affect how the OLLA and DQN algorithms affect the BER performance of communication link scheduling. The OLLA algorithm may be more appropriate if a continuous action space is involved because it can handle the problem of the continuous action space more effectively. However, due to the way the DQN algorithm handles the discrete action problem, it may be a superior fit for the discrete action space. Because the action space for the communication link scheduling problem is discrete, the DQN method may be a preferable choice for scheduling decisions because it performs better on average than the OLLA algorithm in terms of BER. The DQN algorithm is appropriate for the discrete action space problem because it uses knowledge of the Q value function to choose actions that can reduce average BER. The DDQN algorithm is an enhancement to the DQN method that may select the action strategy in the situation of discrete action space more correctly, thereby lowering the average BER even more.

A highly efficient scheduling algorithm can optimize resource allocation, increase anti-interference performance, increase resource utilization, and adapt to dynamic environments, resulting in a significant increase in communication link throughput. A system with high throughput can process data transmission more quickly and boost the network’s data transmission effectiveness. The algorithm’s average BER performance benefits in 5G NR-V2I scenarios are primarily seen in the high dependability, potent anti-interference, self-adaptability, and high throughput it offers. These benefits will improve the efficiency and reliability of 5G vehicle communication, enabling stable and reliable data transmission between vehicles and infrastructure in a challenging wireless communication environment. Figure 8a,b depict the throughput performance of the various methods for delays of 0 μs and 10 μs, respectively, and the effectiveness of the suggested DDQN algorithm at a certain delay. The suggested algorithm is 22 Mbps, 61 Mbps, and 88 Mbps higher than the throughput of the DQN algorithm, OLLA algorithm, and NoOLLA algorithm, respectively, when the delay and frequency bias are 0 μs and 281 Hz. The throughput performance of several methods under doppler shifts of 250 Hz and 500 Hz is shown in Figure 8c,d, respectively. Among these, the suggested DDQN algorithm’s throughput performance at the same multispectral shift is much enhanced. The throughput using the suggested method is 26 Mbps, 51 Mbps, and 78 Mbps higher than the throughput using the DQN algorithm, OLLA algorithm, and NoOLLA algorithm, respectively, when the frequency bias and time delay are 250 Hz and 0 μs, respectively. The OLLA algorithm has the flexibility to optimize local action selection under dynamic channel and network conditions, improve resource consumption efficiency, and increase throughput. If the OLLA algorithm is not used when scheduling the communication connection or if the search in the continuous action space or discrete action space is not efficient or flexible enough, the throughput of the link may be impacted. For the discrete action space problem, the DQN algorithm works better. It is better suited for highly reliable intelligent downlink scheduling in 5G NR-V2I scenarios by learning the Q value function to choose the actions that can maximize throughput. The DDQN algorithm used in this research may better optimize the link resource allocation and increase the throughput of communication lines by lowering the overestimation of the Q value.

Finally, the average iterations of DQN and DDQN are compared. Comparing the average number of iterations helps identify which algorithms converge faster to a suitable performance level under the same training conditions. Fewer iterations usually indicate a more efficient training process. In addition, fewer iterations may mean that the training process is more stable, which also means that the algorithm requires fewer computational resources. As shown in Table 3, although DQN is less than DDQN in the number of iterations, DDQN is more stable when the environment deteriorates, because its number of iterations changes more slowly.

## 5. Conclusions

This article suggests an ultra-reliable intelligent downlink scheduling technique based on DDQN for the 5G NR-V2I autonomous driving scenario. With $D_{\mathrm{CQI}}$, $D_{\mathrm{RI}}$, and $D_{\mathrm{PMI}}$ from the measurement feedback of the vehicle terminal and the statistics $B_{P\text{-}\mathrm{slot}}$ as input variables, this approach combines the DNN network and Q-learning algorithm. The BER for all downstream transmission modalities is the output. According to the Q-learning concept, the RSU chooses the MCS and the number of multiplexing layers with the lowest BER for downlink transmission. In order to avoid imperfection in the learning process or noise in the data that may lead to bias, this paper uses appropriate data preprocessing methods to reduce the impact of noise, such as filtering or smoothing. In this paper, the empirical replay mechanism is used to reduce the problem of high Q overestimation. In order to reduce the cost of two independent networks, this paper adopts some techniques to reduce the training cost, such as sharing some parameters and reducing the network size. In order to avoid a DDQN that may lead to over-exploitation and less exploration, this paper uses appropriate exploration strategies, such as the ε-greedy strategy, to ensure that the algorithm maintains a certain degree of exploration. In order to avoid policy oscillations that may be caused by managing two Q networks, this paper uses a soft update or progressive update to smooth the policy update process. In order to avoid overfitting problems, this paper uses techniques, such as regularization and stopping training in advance, to avoid overfitting.

The simulation demonstrates that the ultra-reliable intelligent downlink scheduling algorithm based on DDQN outperforms the NoOLLA, OLLA, and DQN algorithms in terms of average error rate and throughput performance, ensuring the ultra-reliability and efficiency of communication between vehicles and infrastructure. In addition, although DQN is less than DDQN in the number of iterations, DDQN is more stable when the environment deteriorates, and its number of iterations changes more slowly. In future research, we will consider the use of appropriate state representation methods by using recurrent neural network (RNN) or other timing models to deal with dynamic environments to cope with the training difficulties that may be caused by highly dynamic environments. Considering that the algorithm update under the condition of real-time change may require a lot of computing resources, the use of distributed computing can be considered to improve computing efficiency. In order to ensure the stability of the system quickly adapted to new conditions, a buffer zone or sliding window can be considered to slow down the adaptation speed of the model to maintain the stability of the system.

## Figures and Tables

**Table 1:** Communication system parameter settings.

Parameter Name	Parameter Value
Carrier Frequency	5.925 GHz
Carrier Interval	30 kHz
Number of subcarriers	624
FFT Points	1024
Modulation mode	QPSK, 16 QAM, 64 QAM, 256 QAM
Channel model	TDL
Number of antennas of road test unit	32
Number of vehicle terminal antennas	4
Number of subframes	300

**Table 2:** DQN system training parameter settings.

Parameter Name	Parameter Value
Iteration number	1000
Memory size	1000
Frequency of update of target network parameters	150
Activation function	Tanh
Loss function	Huber
Learning rate	0.01
Batch size	16
Number of vehicle terminal antennas	0.9

**Table 3:** System training duration.

Parameter Name	Performance Name	DQN Average Iterations	DDQN Average Iterations
Time delay = 0 μs	Average BER	732	761
Time delay = 10 μs	Average BER	775	784
Frequency offset = 250 Hz	Average BER	753	789
Frequency offset = 500 Hz	Average BER	794	810
Time delay = 0 μs	Average Throughput/(Mbps)	703	732
Time delay = 10 μs	Average Throughput/(Mbps)	731	747
Frequency offset = 250 Hz	Average Throughput/(Mbps)	726	752
Frequency offset = 500 Hz	Average Throughput/(Mbps)	765	773

## Data Availability

Due to privacy, we can not provide the data.
